# A bispecific T cell engager targeting Glypican-1 redirects T cell cytolytic activity to kill prostate cancer cells

**DOI:** 10.1186/s12885-020-07562-1

**Published:** 2020-12-10

**Authors:** Maria E. Lund, Christopher B. Howard, Kristofer J. Thurecht, Douglas H. Campbell, Stephen M. Mahler, Bradley J. Walsh

**Affiliations:** 1 Glytherix Ltd, Suite 2 Ground Floor, 75 Talavera Road Macquarie Park, Sydney, NSW 2113 Australia; 2 Centre for Advanced Imaging, The University of Queensland, St Lucia, QLD 4072 Australia; 3 Australian Institute for Bioengineering and Nanotechnology, The University of Queensland, St Lucia, QLD 4072 Australia; 4 ARC Training Centre for Biopharmaceutical Innovation, The University of Queensland, St Lucia, 4072 Australia; 5 ARC Training Centre for Innovation in Biomedical Imaging Technologies, The University of Queensland, St Lucia, 4072 Australia

**Keywords:** BiTE, Prostate cancer, Glypican-1, Immunotherapy, Checkpoint inhibitor, Miltuximab^®^

## Abstract

**Background:**

Glypican-1 is a heparan sulfate proteoglycan that is overexpressed in prostate cancer (PCa), and a variety of solid tumors. Importantly, expression is restricted in normal tissue, making it an ideal tumor targeting antigen. Since there is clinical and preclinical evidence of the efficacy of Bispecific T cell Engager (BiTE) therapy in PCa, we sought to produce and test the efficacy of a GPC-1 targeted BiTE construct based on the Miltuximab^®^ sequence. Miltuximab^®^ is a clinical stage anti-GPC-1 antibody that has proven safe in first in human trials.

**Methods:**

The single chain variable fragment (scFv) of Miltuximab^®^ and the CD3 binding sequence of Blinatumomab were combined in a standard BiTE format. Binding of the construct to immobilised recombinant CD3 and GPC-1 antigens was assessed by ELISA and BiaCore, and binding to cell surface-expressed antigens was measured by flow cytometry. The ability of MIL-38-CD3 to activate T cells was assessed using in vitro co-culture assays with tumour cell lines of varying GPC-1 expression by measurement of CD69 and CD25 expression, before cytolytic activity was assessed in a similar co-culture. The release of inflammatory cytokines from T cells was measured by ELISA and expression of PD-1 on the T cell surface was measured by flow cytometry.

**Results:**

Binding activity of MIL-38-CD3 to both cell surface-expressed and immobilised recombinant GPC-1 and CD3 was retained. MIL-38-CD3 was able to mediate the activation of peripheral blood T cells from healthy individuals, resulting in the release of inflammatory cytokines TNF and IFN-g. Activation was reliant on GPC-1 expression as MIL-38-CD3 mediated only low level T cell activation in the presence of C3 cells (constitutively low GPC-1 expression). Activated T cells were redirected to lyse PCa cell lines PC3 and DU-145 (GPC-1 moderate or high expression, respectively) but could not kill GPC-1 negative Raji cells. The expression of PD-1 was up-regulated on the surface of MIL-38-CD3 activated T cells, suggesting potential for synergy with checkpoint inhibition.

**Conclusions:**

This study reports preclinical findings into the efficacy of targeting GPC-1 in PCa with BiTE construct MIL-38-CD3. We show the specificity and efficacy of the construct, supporting its further preclinical development.

**Supplementary Information:**

The online version contains supplementary material available at 10.1186/s12885-020-07562-1.

## Background

Prostate cancer (PCa) remains a leading cause of death amongst men, with an expected 191,930 new cases diagnosed in the United States in 2020 [1], representing 7.1% of global cases [2]. In the United States alone, 33,300 deaths are forecast for 2020 [1]. Currently, depending on the stage of disease, treatment options include active surveillance (sometimes for many years), prostatectomy, androgen deprivation therapy, chemotherapy, radiotherapy or a combination of these [3]. Despite therapy many patients ultimately develop androgen-insensitive PCa metastatic castrate resistant prostate cancer (mCRPC). At this late stage, median survival is less than 3 years [4]. There is a crucial need for novel effective therapies for PCa.

Bispecific T cell engaging antibodies (BiTEs) show great promise in cancer therapy. BiTEs are able to redirect tumour ignorant T cells to specifically lyse tumour cells in the absence of antigen presentation, therefore overcoming tumour cell evasion tactics such as down-regulation of MHC expression. Blinatumomab, targeting CD19 and CD3, has seen clinical success in the treatment of Philadelphia chromosome-negative relapsed/refractory B cell precursor Acute Lymphocytic Leukemia and received FDA approval in 2014. Since then, there has been a wave of bispecific T cell-engaging constructs in pre-clinical and clinical development, varying in their structure, function and target antigen, designed both for haematological and solid tumours [5, 6]. In prostate cancer, a PSMA targeting BiTE, Pasotuxizumab, showed good efficacy in a preclinical animal model of PCa [7, 8]. The molecule is now in Phase I for mCRPC, where some clinical efficacy has been demonstrated (NCT01723475) [9]. Despite these exciting results demonstrating the clinical potential of a BiTE construct in mCRPC, additional therapies are needed as PSMA is not expressed by all PCa patients [10]. Moreover, PSMA is expressed in a number of normal tissues, including moderate expression in the salivary glands, which has led to side effects such as xerostomia in PSMA-targeted radiotherapy studies [11]. These data suggest there is a need for novel BiTE targets for PCa that exhibit an improved expression profile.

Glypican-1 (GPC-1) is a heparan sulfate proteoglycan consisting of a core protein linked to several heparan sulfate chains and attached via a GPI anchor to the cell surface [12]. Playing a critical role in tumour cell growth, invasion and metastasis as a growth factor co-receptor, GPC-1 is overexpressed in a variety of solid tumours, including PCa [12, 13]. In line with the role of GPC-1 in tumour progression, expression of GPC-1 increases with increasing Gleason Grade [13], suggesting its potential as a target for aggressive, difficult to treat lesions, including in mCRPC. Importantly, GPC-1 is not expressed in normal adult tissue [13, 14], indicating that targeting of GPC-1 may be associated with an improved side effect profile as compared to other antigens. Indeed, several preclinical studies have examined the effect of targeting GPC-1 in animal models showing no adverse effects [15, 16]. Moreover, a first in human clinical trial using the chimeric anti-GPC-1 antibody Miltuximab^®^ (Glytherix Ltd) was shown to be safe and well tolerated, exhibiting no drug related adverse events [17].

Given the strong rationale for the targeting of GPC-1 using a BiTE construct in PCa, we engineered Miltuximab^®^ as a CD3 engaging BiTE. This BiTE demonstrated effective tumour cell killing in PCa cell lines DU-145 and PC3. The release of pro-inflammatory cytokines from T cells activated by MIL-38-CD3 was also observed. An up-regulation of PD-1 on the surface of the activated T cell population suggested the potential for synergy of MIL-38-CD3 therapy with checkpoint inhibition.

## Methods

### Cell lines

DU-145 and PC3 cells were purchased from ATCC. Jurkat and Raji cells purchased from ATCC, were a generous gift from Dr. Andrew Hutchinson. All cell lines were tested for lineage and mycoplasma contamination quarterly (CellBank Australia, Sydney). DU-145 and Jurkat cells were cultured in Roswell Park Memorial Institute medium (RPMI; Life Sciences) supplemented with 10% v/v Foetal Bovine Serum (FBS; Interpath). PC3 cells were cultured in RPMI supplemented with 20% v/v FBS. Raji cells were cultured in RPMI supplemented with 10% v/v heat inactivated FBS (HI FBS; 56 °C, 30 min).

### MIL-38-CD3 BiTE

A gene sequence encoding the MIL-38-CD3 bispecific antibody was generated by Geneart (Life Technologies). The MIL-38-CD3 construct was designed as a tandem scFv consisting of anti-GPC-1 scFv (Miltuximab^®^ sequence; GTL Ltd.) linked to anti-CD3 scFv (derived from Blinatumomab sequence; Micromet; http://www.drugbank.ca/drugs/DB09052) by a glycine serine linker. A signal peptide was included to enable secretion of the construct from Chinese Hamster Ovary (CHO) cells into the cell culture medium [18]. A 6xHis tag was included at the N-terminal of the sequence for purification, and a c-myc tag at the C-terminal (EQKLISEEDLN) was also included as a detection tag for Western blotting, ELISA and flow cytometry.

Additional control constructs were designed including

1. CD3-PEG as a positive control for CD3 binding. The construct targets CD3 and polyethylene glycol (PEG) moeity as a target not expressed in human cells
2. J591-PEG as a negative control for CD3 binding with one arm specific for PSMA (J591 sequence), and one arm specific for PEG, thus not binding CD3.

Both constructs contained anti-PEG gene sequences from patent US20120015380A1 which are used by our UQ collaborators for targeted delivery of PEGylated nanomaterials to cancer and immune cells [18]. CD3 sequences were derived from Blinatumomab sequence and J591 (anti-PSMA) Vh and Vl gene sequences were derived from the patent WO2003024388A2.

The MIL-38-CD3 and other construct genes were cloned into the pcDNA 3.1 mammalian expression vector using HindIII and Not1 restriction sites and standard ligation based cloning methodology and confirmed by DNA sequencing at the Australian Genome Research Facility (AGRF). Larger quantities of DNA required for transient transfections were prepared from 400 ml of transformed *E. coli* (top10) using DNA Midiprep kits (Macherey Nagal). Final precipitated DNA was resuspended in 1 ml of sterile water, concentration determined at A260 using Nanodrop 1000 and stored at − 20 °C for transient transfections.

Suspension-adapted CHO cells were transiently transfected with DNA encoding MIL-38-CD3 (and other BiTE controls) using PeiMax transfection reagent (a total of 3 × 10^8^ cells in 100 ml; 3 × 10^6^ cells/ml). Transfected cells were cultured for 12–14 days supplemented with specialised feed medium (starting density 1-2 × 10^6^ cells/ml, final density 6 × 10^6^ cells/ml), at a temperature of 32 °C, 7.5% CO_2_ with shaking at 130 rpm. The expression and secretion of proteins of interest in the medium was confirmed by western blotting using the HRP labelled anti-myc antibody (Miltenyi Biotec; clone SH1-26E7.1.3) to detect the C-terminal myc tag. This also confirmed expression of full length protein. Transfections were stopped when cell viability, measured by trypan blue staining was below 60%. The cells were then centrifuged at 4750 rpm for 10mins and the supernatant was collected and filtered through a 0.2 μm PES filter (Millipore). The filtered supernatant was loaded at 5 ml/min over a 5 ml HisTrap Excel column (GE Healthcare; Australia), equilibrated in 20 mM sodium phosphate, 500 mM NaCl pH 7.4. The bound protein was eluted in 15 ml of 20 mM sodium phosphate, 500 mM NaCl 500 mM Imidazole pH 7.4 buffer, and buffer exchanged into 20 mM sodium phosphate, 500 mM NaCl pH 7.4 using a HiPrep 26/10 Column (GE Healthcare; Australia). Protein concentration was determined by NanoDrop at Abs 280 nm and the extinction coefficient of the protein.

### In vitro characterization of MIL-38-CD3

#### Flow cytometry

Cells were collected from culture and incubated with MIL-38-CD3 (10 μg/ml) for 45 min on ice. Cells were washed 3 times with PBS/2% HI FBS (Flow cytometry staining wash; FSW) then stained with anti-cmyc-FITC (Miltenyi Biotec, Australia; clone SH1-26E7.1.3) or anti-his FITC or PE (Miltenyi Biotec, Australia; clone GG11-8F3.5.1) for 45 min on ice. Cells were washed 3 times in FSW and then acquired on the BD Fortessa X20 (BD, Australia) using FACS DIVA software (BD, Australia). The viability dye topro-3-iodide (Life Technologies; Australia) was used to restrict analysis to viable cells. This dye was added to a final concentration of 1 μM immediately prior to acquisition. Data were analysed in FCS Express V5 (De Novo Software, USA).

#### ELISA

The MIL38-CD3 construct alongside other controls were tested for binding to recombinant human GPC-1 (Rnd Systems) and human CD3 delta and CD3 epsilon (Abcam) using ELISA. For ELISA, Maxisorp plates (Nunc) were coated with 1 μg/ml of rGPC-1 or hCD3 diluted in PBS for 20 h at 4 °C. Each well was blocked with 200 μl 2% skim milk in PBST (PBS+ 0.05% Tween 20) for 1 h at room temperature. The blocker was then decanted and 100 μl of test construct samples (supernatant or purified sample) were added to wells. Purified J591-PEG (binds to PSMA and PEG) was used as a negative control. CD3-PEG (binds hCD3 and PEG) was used as a positive control for CD3 binding. The samples and controls were incubated for 2 h at room temperature, then washed 3 times with PBST (200 μl per wash), then 100 μl HRP labelled anti-c-myc antibody (Miltenyi Biotech, diluted 1/5000 in block solution) was added to plates for 1 h, then the plate was washed 3 times with PBST. The substrate TMB (Sigma; 100 μl per well) was added to wells and incubated for 15mins in the dark to yield a colorimetric reaction. The reaction was stopped with 2 M sulphuric acid (100 μl per well) and absorbances were recorded at A450nm using the SpectraMax plate reader.

#### Western blot

Protein samples were heated at 95 °C for 5 mins in NuPAGE sample loading buffer (Invitrogen) with DTT reducing agent (Invitogen) and run on a 4–12% Bis-Tris PAGE (Invitrogen) in 1xMES Buffer for 30mins at 200 V. Proteins were transferred to PVDF membranes using Transblot System (Biorad) set on a mixed size program for 7 mins. Membranes were blocked in 2%MILK-PBST for 1 h at room temp. Ten milliliter of HRP labelled anti-c-myc antibody (Miltenyi Biotech, diluted 1/5000 in block solution) was added to membranes for 1 h, then membranes were washed in PBST 3x5mins. Ten milliliter of ECL reagent (Invitrogen) was added to the membrane for 2 mins and chemiluminesence visualised using the Biorad Gel Doc System.

### BiaCore analysis

The determination of binding and affinity of MIL38-CD3 construct for GPC1 and CD3 was determined using the Biacore T200 platform in collaboration with the National Biologics Facility (NBF).

#### Immobilisation pH scouting for GPC-1

For initial immobilisation pH scouting experiments recombinant human GPC-1 (R&D Systems) was diluted 1 in 50 in acetate buffer, pH 4, 4.5, 5, 5.5 to 100 μg/ml and tested for binding to CM5 chip surface. Binding was similar under all conditions, and so pH 5.5 was chosen since it is a milder buffer. 3700RU of GPC-1 was immobilized on flowcell and compared to blank flowcell. MIL38-CD3 construct was 2x serially diluted from 50 μg/ml to 0.375 μg/ml (8 dilutions in total) and injected for 30s at 30 μl/min. 2000 RU bound to the GPC-1 derived surface when 50 μg/ml test construct was injected. Both 3 M MgCl2 and 10 mM Glycine-HCL pH 1.7 were tested as regeneration buffer and it was found that 10 mM glycine was able to regenerate the chip surface well so this was used for the kinetics experiments.

#### MIL-38-CD3 to GPC-1 kinetics

For the kinetics study, 100RU of GPC-1 was immobilized on the flowcell, and compared to a second flow cell as blank control. Five concentrations of MIL-38-CD3 were prepared for injection, 50 μg/ml, 16.67 μg/ml, 5.55 μg/ml, 1.85 μg/ml, 0.62 μg/ml. Chips were regenerated with 10 mM glycine at pH 1.7. Data analysis was generated using 1:1 model.

#### CD3-fc to MIL-38-CD3 kinetics

MIL-38-CD3 was captured on GPC-1 immobilised on the flowcell and 5 concentrations of human CD3-fc were prepared for injection, 50 μg/ml, 16.67 μg/ml, 5.55 μg/ml, 1.85 μg/ml, 0.62 μg/ml. 10 mM glycine at pH 1.7 was used as regeneration buffer. Data was analysed using both a bivalent analyte model and a steady state affinity model.

### Preparation of purified peripheral blood T cells

Human buffy coats were obtained from the Australian Red Cross Life Blood under a Material Supply Agreement. The Australian Red Cross Life Blood obtained consent for the use of donor samples, and the use of these samples for the current study was approved by the Australian Red Cross Life Blood (approval number 17-05NSW-13). Human ethics approval was obtained from Macquarie University Human Ethics Committee (approval number 5201700069). To obtain the peripheral blood mononuclear cell (PBMC) fraction, buffy coats were diluted 1:1 in room temperature (RT) RPMI and then 4 ml of diluted buffy coat was overlayed on 3 ml Ficoll Paque (RT; GE Healthcare, Australia). Overlays were centrifuged at 400×g, 35 min, with brakes and acceleration off. The plasma layer at the top was removed by pipetting using a transfer pipette, and the leukocyte layer at the interface of the ficoll and the plasma was removed to a new 15 ml falcon tube. Cells were topped up with 10x volume of RT RPMI and centrifuged at 400×g, 20 min to collect. Cell pellets from the same donor were combined and cells were then resuspended in 10 ml RPMI and centrifuged at 150×g for 10 min to remove platelets. The supernatant was carefully poured off and PBMCs were counted. PBMCs were resuspended in HI FBS (90% v/v) + DMSO (10% v/v) at 1-3 × 10^7^ cells/ml/tube for storage and aliquoted into cryovials. Cryovials were frozen in CoolCells or isopropanol at − 80 °C and then moved to liquid nitrogen at 24 h post freezing. To resuscitate PBMCs, cells were thawed rapidly in a water bath and immediately rinsed with pre-warmed (37 °C) RPMI supplemented with HI FBS (10% v/v; PBMC media) at 300×g, 5 min. To enrich for T cells by negative selection, the human Pan T cell isolation kit II (Miltenyi Biotec, Australia) was used according to the manufacturer’s instructions.

### In vitro T cell activation assays and phenotyping

Enriched T cells were prepared at densities of 2 × 10^6^ cells/ml in PBMC media. DU-145 cells were collected by rinsing with PBS and then incubation with PBS/EDTA 2 mM for 15 min at 37 °C/5%CO_2_. Cells were then prepared at a density of 2 × 10^5^ cells/ml in PBMC media. Constructs (MIL-38-CD3 or control BiTE CD3-PEG, were prepared at 11x final concentration for addition to the plate in PBMC media. For positive control of T cell activation, 96 well flat bottomed tissue culture plates (Falcon; FAL353072) were coated with anti-CD3 on some wells. Anti-human CD3 functional grade (EBioscience; OKT3; 16–0037-85; lot 4,308,895; 5 μg/ml diluted in sterile PBS) was coated on wells of the plate for 2 h at 37 °C or O/N at 4 °C. The wells were then washed twice (200 μl) with sterile PBS. Construct (MIL-38-CD3 or control BiTE) were plated first (10 μl), followed by test cell line (50 μl; 1 × 10^4^ cells) and PBMCs or T cells (50 μl; 1 × 10^5^ cells) to a final Effector:Target ratio of 10:1. Cells were incubated for 24 h for measurement of CD69 and CD25 levels or 72 h for cytokine release assays, in culture (37 °C/5%CO_2_).

To measure CD69 and CD25 levels, cells were collected by gentle scraping and stained with a cocktail of antibodies against CD3 (BD; UCHT1), CD4 (BD; RPA-T4), CD8 (BD; RPA-T8), CD25 (BD; M-A251), CD69 (BD; FN50) for 45 min on ice, protected from light. Cells were then washed three times with FSW and resuspended in FSW before addition of viability dye Topro-3-Iodide (Life Technologies, Australia).

To measure cytokine release, culture supernatants were collected after centrifugation of the plate at 72 h (300×g, 5 min). Supernatants were frozen at − 20 °C until analysis. For analysis, 50 μl cell supernatant was measured using the human TNF ELISA kit (BD, Australia) or human IFN-γ ELISA it (BD, Australia), according to manufacturer’s instructions.

For measurement of PD-1 expression, T cells were co-cultured with DU-145 tumour cells and MIL-38-CD3 for 48 h, before T cells were harvested by gentle scraping and pelleted (300×g, 5 min). Cells were incubated with anti-PD-1-PE (BD; clone M1H4) for 45 min on ice, then washed three times and resuspended in FSW, before addition of viability dye topro-3-iodide (Life Technologies, Australia). Expression of PD-1 was then measured by acquisition on the BD Fortessa X20 (BD, Australia).

### In vitro cell killing assays

Target tumour cells were labelled with Vybrant DIO (Life Technologies, Australia) for 18 min in RPMI at 37 °C in a 15 ml falcon tube. Cells were then centrifuged (180×g, 5 min for DU-145 and C3 cells, or 300×g, 5 min for Raji) and the supernatant was discarded. Enriched T cells were resuspended in 10 ml PBMC media and centrifuged. Cells were then prepared at a density of 4 × 10^5^ cells/ml in PBMC media. Test antibody construct was plated in 96 well flat bottomed tissue culture plates in a volume of 10 μl at the appropriate concentration, followed by 50 μl tumour cell suspension, followed by 50 μl T cell suspension. Cells were incubated for 72 h. The plate was centrifuged (300×g, 5 min), and the supernatant removed by pipetting and discarded. The plate was then washed twice with PBS (200 μl/well; 300×g, 5 min) and each time the supernatant was discarded. Cells were detached from the plate by incubation with Trypsin 0.25% EDTA in RPMI (Sigma Aldrich, Australia) for 3 min at 37 °C/5%CO_2_. Detachment of cells was confirmed by microscopy. Trypsinisation was stopped by addition of an equivalent amount of R10 containing Topro-3-iodide (Life Technologies, Australia; 0.3 μM; final concentration per well 0.15 μM) to the plate. The plate was then kept on ice until analysis. Cells were then acquired on the BD Fortessa X20 (BD, Australia) and analysis was performed in FCS Express Version 5 (De Novo Software, USA). For analysis, cells were first gated on forward and side scatter to exclude debris, but to include dead cells. Single cells were identified using forward scatter height and area. Analysis was then restricted to FITC^+^ tumour cells, and then Topro-3-Iodide (APC) signal within the tumour cell gate was measured as a proportion of tumour cells.

### Statistics

All statistical tests between two groups were performed using a two-tailed unpaired t test in GraphPad Prism 5.0.

## Results

### Expression and purification of MIL-38-CD3 BiTE

**Fig. 1:**
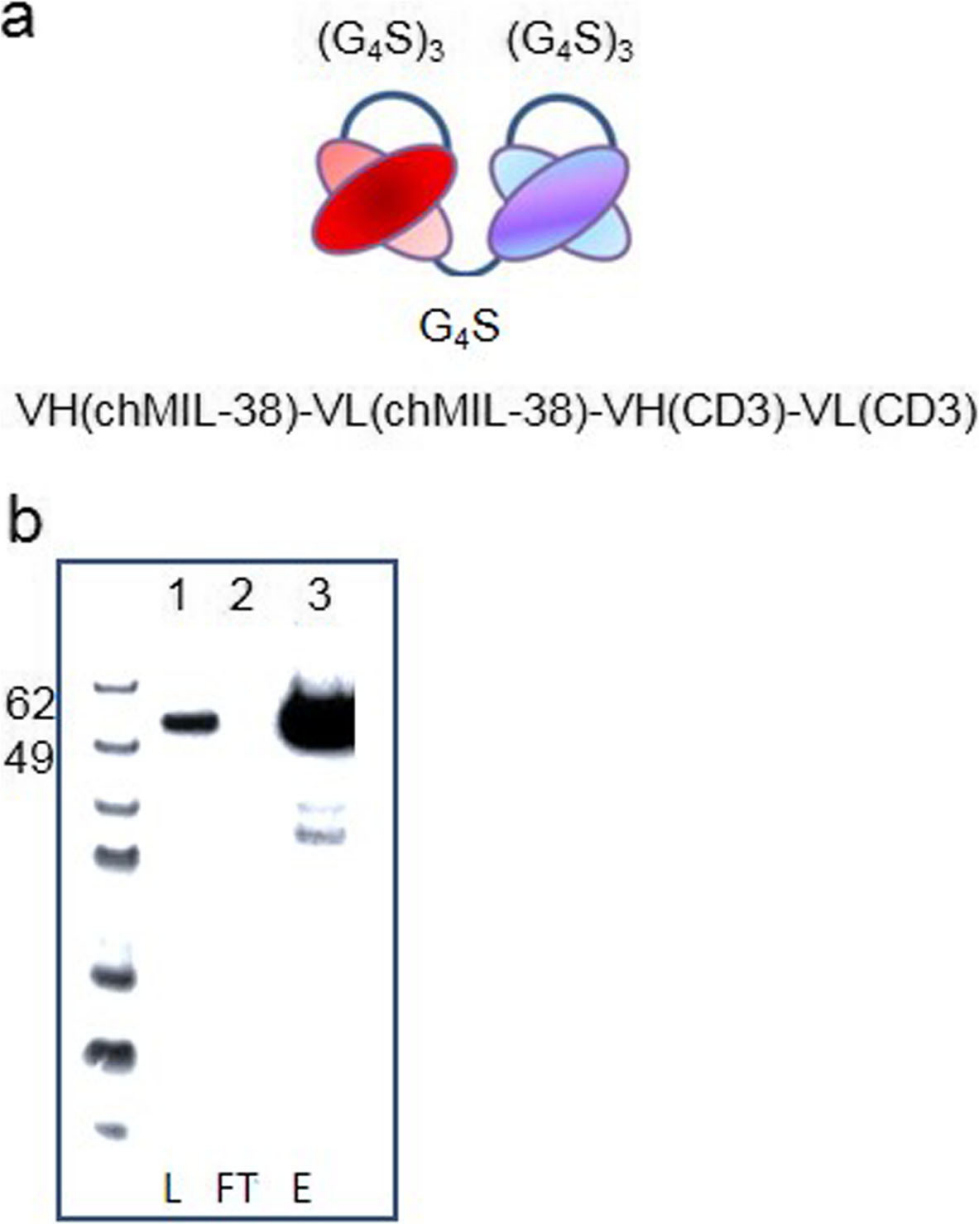
MIL-38-CD3 BiTE Construct. **a** Schematic of MIL-38-CD3 BiTE (**b**) Western blot detection of purified MIL-38-CD3 BiTE (Lane 1: L = supernatant loaded onto purification column, Lane 2: FT = flow through and Lane 3: E = column eluate)

A flexible serine-glycine linker was used to link the MIL-38 scFv to the CD3 scFv, and a 6xHis tag and c-myc tag for purification and detection, respectively, were included (Fig. 1a). The construct was purified using a HisTrap column, and the purity was estimated at > 90% based on lack of detectable product identified in the column flow through by Western blot (Fig. 1b). The size of the construct was estimated at 55 kDa by Western blot (Fig. 1b).

### MIL-38-CD3 BiTE binds to recombinant and cell surface GPC-1 and CD3

**Fig. 2:**
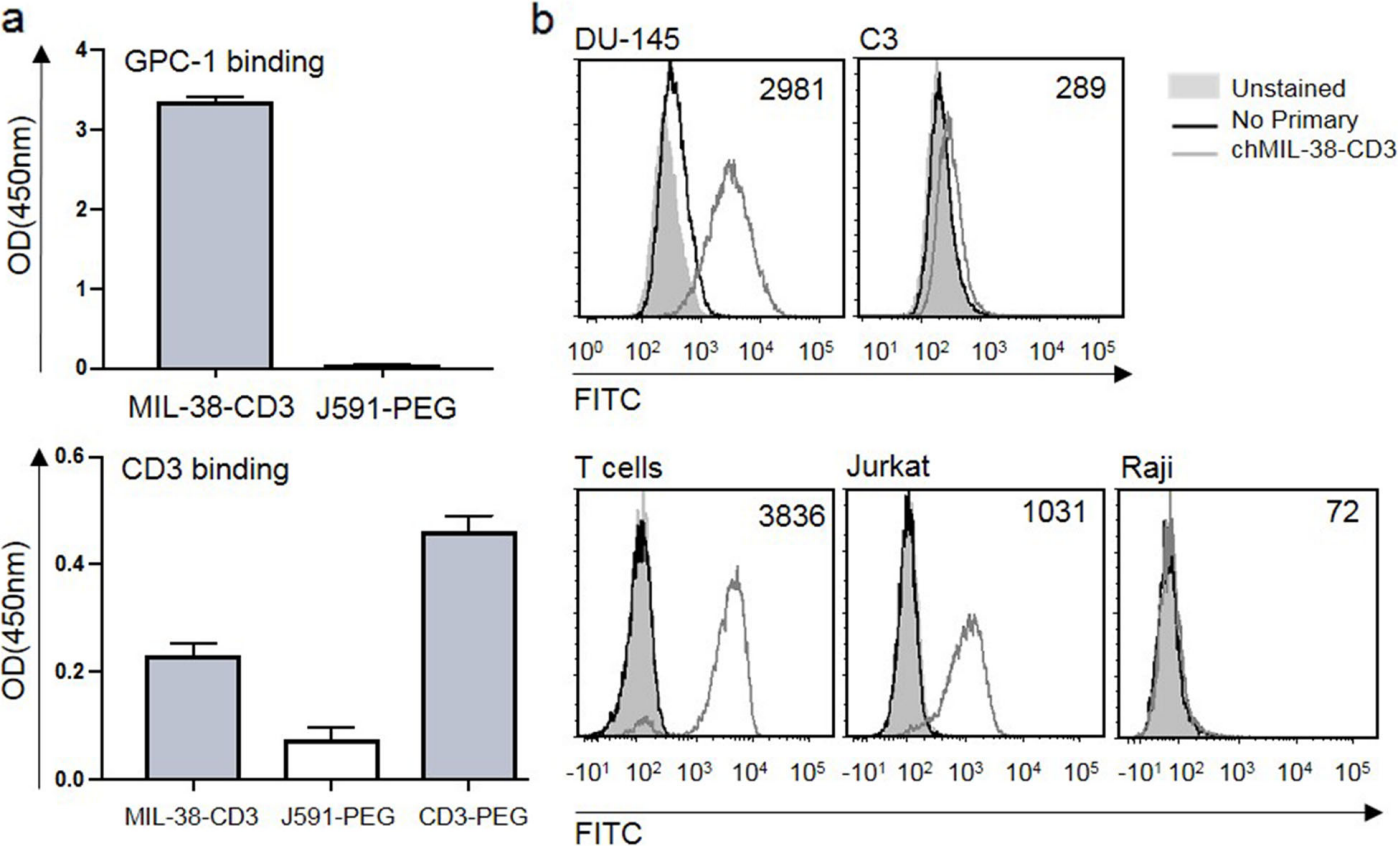
Binding of MIL-38-CD3 BiTE to native cell expressed antigen. **a** Binding of MIL-38-CD3 BiTE and controls J591-mPEG and CD3-mPEG to plate bound recombinant GPC-1 and CD3. **b** Binding of MIL-38-CD3 BiTE to GPC-1 expressed on DU-145 cells and C3 cells was measured by flow cytometry. Binding to CD3 on purified human T cells Jurkat and Raji

The binding of MIL-38-CD3 to recombinant antigen was tested by ELISA. MIL-38-CD3 was found to bind to plate-bound recombinant GPC-1, whereas the control BiTE construct, J591-PEG showed no binding (Fig. 2a). In a similar manner, binding of MIL-38-CD3 to plate-bound CD3 was observed, whereas no binding was observed for control construct J591-PEG. As expected, binding of the positive control BiTE CD3-mPEG, with one arm specific for CD3, showed binding to CD3. Binding kinetics as determined by BiaCore revealed an equilibrium binding constact (kD) of 1 nM for GPC-1 binding, and 1 μM for CD3.

Next, binding of MIL-38-CD3 to cell surface expressed GPC-1 and CD3 was investigated. To assess binding to GPC-1 the DU-145 prostate cancer cell line was used, as this line is known to express approximately 70,000 GPC-1 proteins on the cell surface (as measured by quantitative flow cytometry; Supplementary Figure 1). Two negative control cell lines were chosen that expressed differing densities of GPC-1 on their surface; the B cell lymphoma Raji (261 GPC-1 molecules per cell), and bladder cancer line C3 (approximately 3500 molecules per cell; Supplementary Figure 1). Binding to DU-145 was observed, while binding to C3 cells was detectable but low (Fig. 2b), indicating a dependence of GPC-1 expression for binding. Binding to cell surface CD3 was confirmed using purified peripheral blood T cells, the T cell lymphoma cell line Jurkat, and as a negative control the Raji cell line (Fig. 2b).

### Specific activation of T cells by MIL-38-CD3 BiTE

**Fig. 3:**
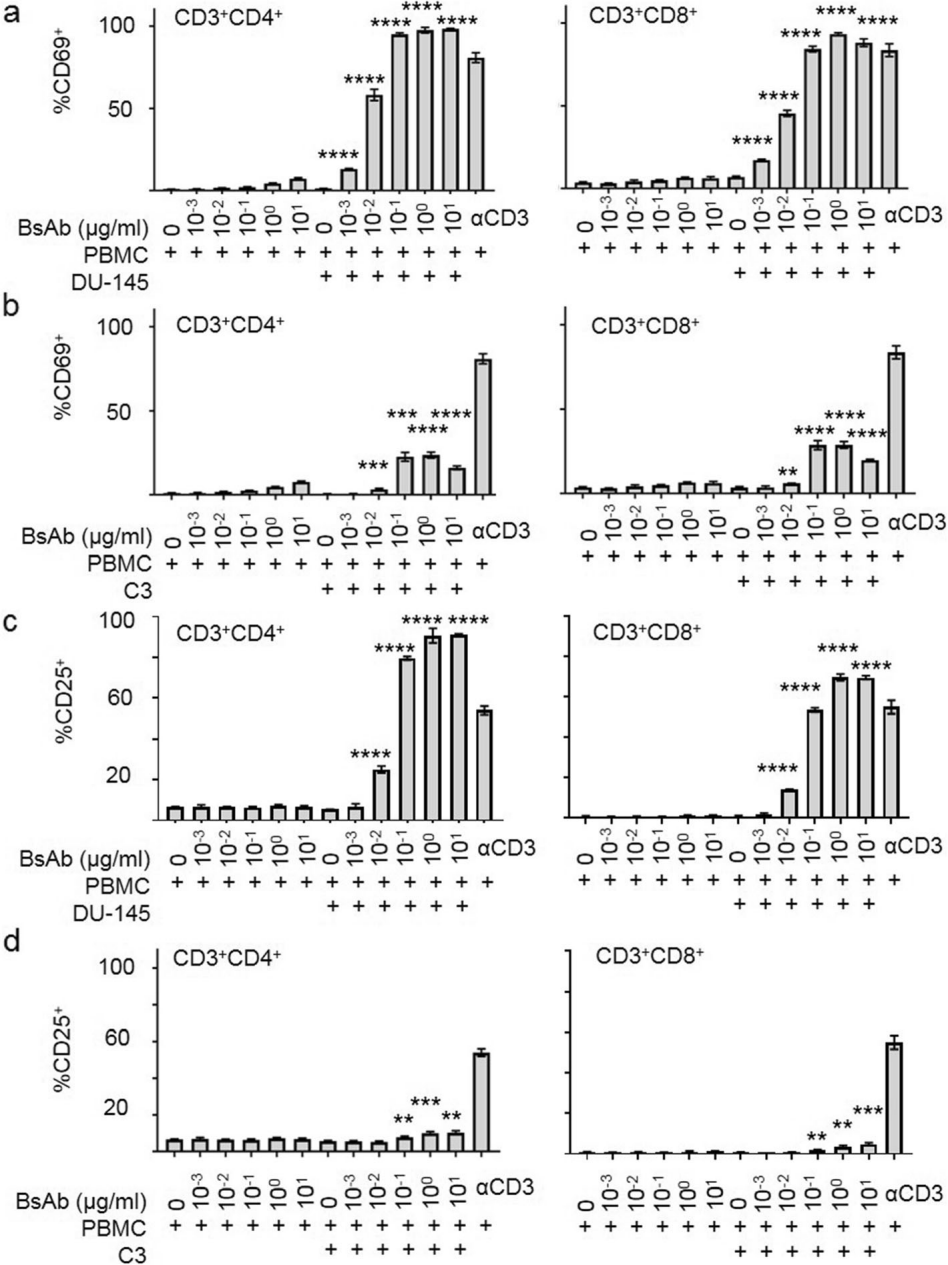
Induction of T cell activation by MIL-38-CD3 BiTE. T cell activation was measured by flow cytometry analysis of CD69 (**a**) or CD25 (**c**) in T cells (within PBMCs) cultured with MIL-38-CD3 BiTE and GPC-1^high^ DU-145 cells within the CD3^+^CD4^+^ or CD3^+^CD8^+^ T cell gates (**a**) or GPC-1^low^ C3 cells (**b**: CD69; **d**: CD25) at a ratio of 10:1 PBMC:tumour cell for 24 h. Data are representative of more than 2 independent experiments. ****p < 0.0001; ***p < 0.001; **p < 0.01

The ability of MIL-38-CD3 BiTE to mediate the activation of T cells in the presence of GPC-1-expressing prostate tumour cells was measured by flow cytometry analysis of the early activation marker CD69 and the late activation marker CD25. The DU-145 cell line was chosen as a line representative of differentiated adenocarcinoma. It is an androgen insensitive line derived from a brain metastasis, that does not express prostate specific antigen (PSA), and is commonly used to model PCa in vitro and in animal studies [19]. Peripheral blood T cells were purified from healthy donors and cultured with varying concentrations of MIL-38-CD3 BiTE and GPC-1^high^ DU-145, or GPC-1^low/negative^ line C3. As expected, both CD4^+^ and CD8^+^ T cell populations cultured with positive control, plate-bound anti-CD3 antibody, became activated (Fig. 3a-d). In the presence of DU-145 cells, at concentrations of MIL-38-CD3 BiTE increasing over two orders of magnitude from 0.001 μg/ml, both CD4^+^ and CD8^+^ subsets, were activated, by measurement of CD69 compared to control (MIL-38-CD3 0 μg/ml) cells (Fig. 3a). Activation, as measured by CD25 up-regulation, was achieved at concentrations at, or greater than, 0.01 μg/ml for both subsets (Fig. 3c). Importantly, there was only minimal increase in T cell activation with any concentration of MIL-38-CD3 BiTE tested in the absence of tumour cells, demonstrating the requirement for antigen recognition (Fig. 3a, c). To confirm the absence of GPC-1 expression within the effector cell population, flow cytometry was used to measure GPC-1 in T cells, showing no expression (Supplementary Figure 3). Moreover, only low levels of T cell activation were seen when the GPC-1^low^ cell line C3 was used, for both populations of T cells (CD4^+^ and CD8^+^; Fig. 3b, d), demonstrating a reliance on engagement of GPC-1 and GPC-1 antigen density for activity. The effect of a non-targeted control antibody CD3-PEG BiTE on T cell activation in the presence of DU-145 cells did not induce T cell activation (Supplementary Figure 2).

### Lysis of GPC-1-expressing tumour cells by MIL-38-CD3 BiTE activated T cells

To assess the ability and specificity of MIL-38-CD3 to induce T cell killing of GPC-1-expressing prostate tumour cells, we chose two clinically relevant prostate cancer cell lines for assessment of T cell killing. Killing of the GPC-1 high expressing DU-145 cell line, closest to adenocarcinoma, was compared to killing of the PC3 cell line, which expresses moderate levels of GPC-1 (20,268 molecules/cell; Supplementary Figure 1). This androgen independent line is derived of a bone metastasis, and is considered closest in phenotype to the more aggressive prostatic small cell neuroendocrine carcinoma [19, 20]. This comparison was also of interest given the varying levels of GPC-1 between these two lines. Firstly, binding of MIL-38-CD3 to PC3 cell line was confirmed by flow cytometry, showing less binding than DU-145, in line with PC3 moderate GPC-1 expression (Supplementary Figure 4a). Then, specific T cell activation in the presence of PC3 cells and MIL-38-CD3 (but not control BiTE CD3-PEG) was confirmed by cytokine release assay (release of IFN-γ from the T cell population; achieved at concentrations of MIL-38-CD3 > 100 ng/ml; Supplementary Figure 4b).

**Fig. 4:**
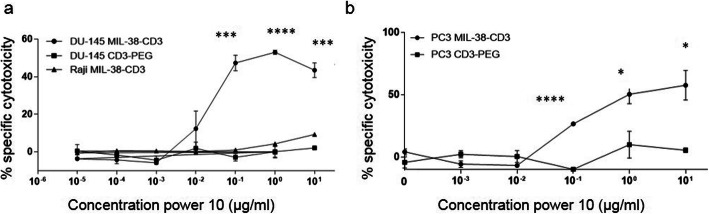
Cytotoxicity within the GPC-1 expressing tumour cell population, mediated by MIL-38-CD3 BiTE. **a**. Fluorescently labelled DU-145 or Raji cells were incubated with MIL-38-CD3 BiTE or control BiTE CD3-PEG together with enriched T cells at a ratio of 10:1 T cell:tumour cell for 72 h. The proportion of dead cells was determined by topro-3-iodide uptake, by flow cytometry. **b**. Labelled PC3 cells were incubated with MIL-38-CD3 and T cells over 72 h and the proportion of dead cells was determined in the same way. ****p < 0.0001; ***p < 0.001, *p < 0.05

To assess tumor cell killing, the GPC-1^high^ DU-145, GPC-1^moderate^ PC3 cells or GPC-1^negative^ Raji cells were cultured at a ratio of 10:1 with unstimulated enriched peripheral blood T cells, together with varying concentrations of MIL-38-CD3 BiTE or non-targeted control BiTE CD3-PEG, for 48 h. Significant cell death was mediated by concentrations 100 ng/ml and greater for DU-145 cells (*p* < 0.001; Fig. 4a). No cell death was observed when DU-145 cells were cultured with CD3-PEG BiTE (Fig. 4a). Importantly, cell death was reliant on GPC-1 expression and MIL-38-CD3 binding, as no cell killing was observed for Raji cells (Fig. 4a), that did not bind MIL-38-CD3 (Fig. 2b). Similarly, in GPC-1 expressing PC3 cells, binding of MIL-38-CD3 was demonstrated (Supplementary Figure 4a) and a significant increase in cell killing was observed at concentrations of 100 ng/ml and greater (*p* < 0.0001; Fig. 4b). These data collectively demonstrate the specificity of the MIL-38-CD3 BiTE and reliance on the recognition of GPC-1 and MIL-38-CD3 binding for activity and demonstrate the efficacy of MIL-38-CD3 in two phenotypically and clinically distinct prostate cancer cell lines.

### Release of inflammatory cytokines by MIL-38-CD3 activated T cells

**Fig. 5:**
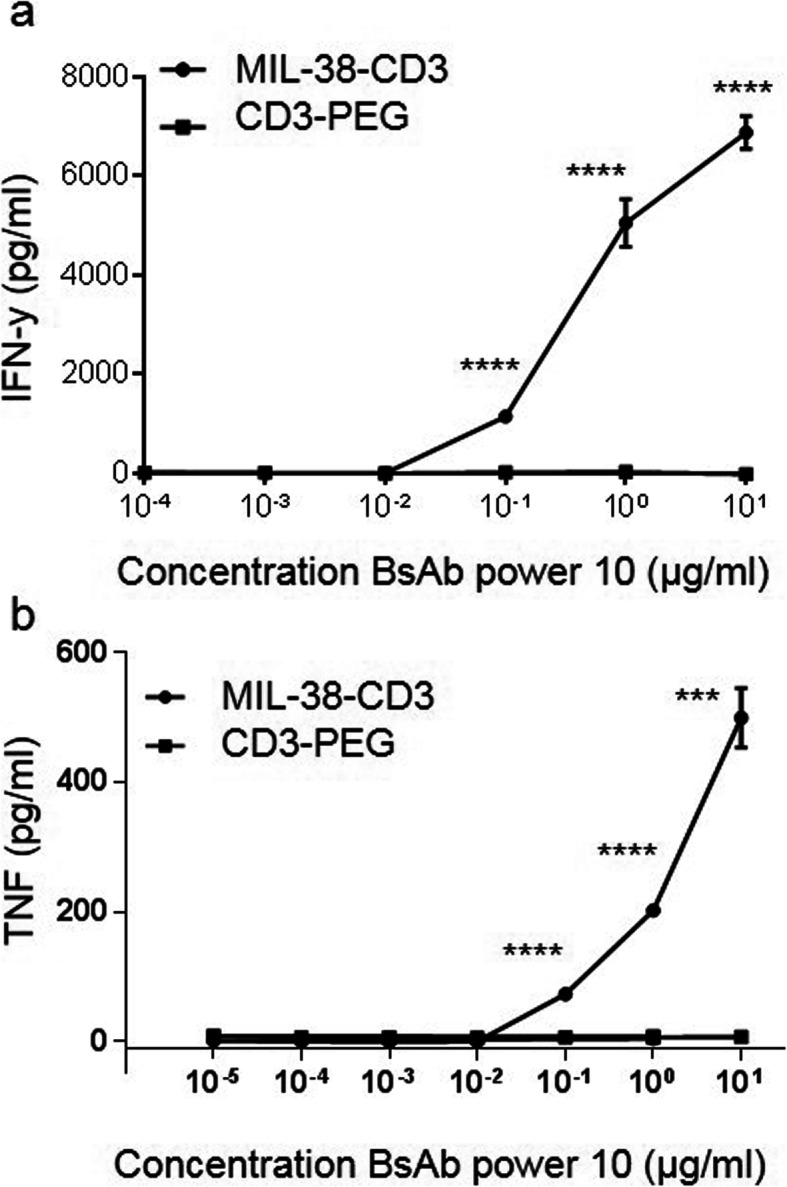
T cell inflammatory cytokine release in response to specific MIL-38-CD3 BiTE activation. Purified T cells were cultured with DU-145 cells in the presence of varying concentrations of MIL-38-CD3 or equivalent concentration of non-targeted control BiTE CD3-PEG for 72 h. Supernatants were collected and assayed for IFN-γ (**a**) and TNF (**b**) ****p < 0.0001; ***p < 0.001

The presence of inflammatory cytokines such as IFN-γ and TNF in the tumour microenvironment is known to promote the recruitment of inflammatory immune cells, activate local immune cells, and promote tumour cell death [21, 22]. Thus, we sought to measure the release of inflammatory cytokines from T cells in response to MIL-38-CD3 in the presence of DU-145 cells. We found significantly increased release of both IFN-γ and TNF when T cells were exposed to DU-145 cells and MIL-38-CD3 BiTE at concentrations of 100 ng/ml and greater of MIL-38-CD3 BiTE (*p* < 0.0001; Fig. 5). Importantly, no cytokine release (for either cytokine) was observed for the control BiTE (Fig. 5).

### T cell up-regulation of PD-1 following MIL-38-CD3 BiTE activation

**Fig. 6:**
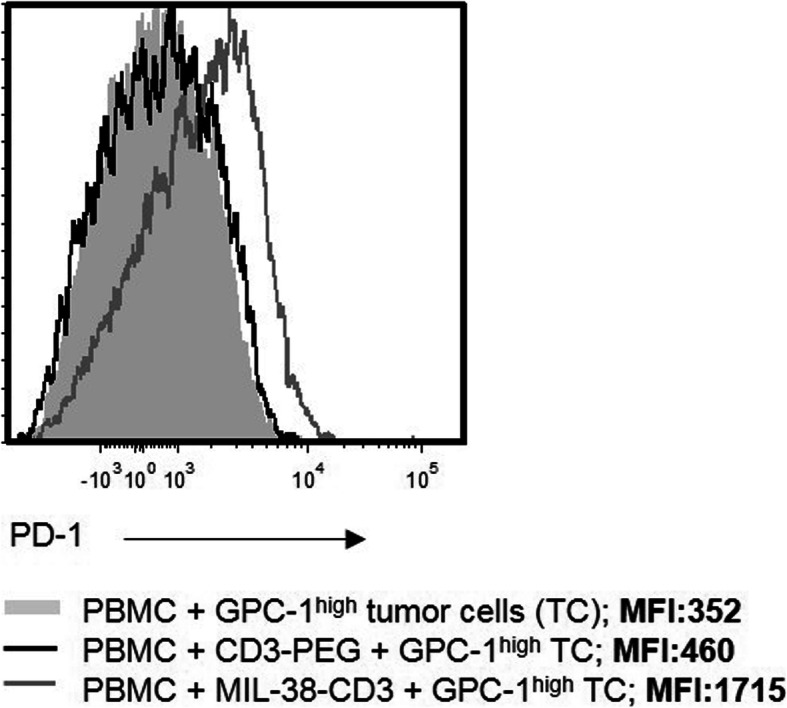
Expression of PD-1 in T cells following specific activation with MIL-38-CD3 BiTE. T cells were cultured with MIL-38-CD3 or a non-targeting BiTE CD3-PEG for 48 h, when PD-1 expression was measured within the CD3^+^ population by flow cytometry

Checkpoint inhibitor therapeutics have thus far proven variable in efficacy in solid tumours, particularly those that are considered less immunogenic, for example, in PCa [23]. One therapeutic approach of interest to us is the combination of BiTE therapy with checkpoint inhibition. Indeed, it is foreseeable that BiTE mediated recruitment and activation of T cells at the tumour site could be perpetuated by checkpoint inhibition. Thus, to establish the potential utility of a checkpoint inhibitor in this system, we measured PD-1 expression in T cells specifically activated by MIL-38-CD3 BiTE. An increase in expression of PD-1, following culture of T cells with DU-145 prostate tumour cells was observed, but no increase was seen when using the non-targeted control BiTE, CD3-PEG (Fig. 6). Thus, there may be rationale for improved BiTE efficacy with checkpoint inhibition.

## Discussion

The overexpression of GPC-1 in PCa, and its restricted expression in normal adult tissue, make GPC-1 an attractive therapeutic target in an indication where there is an unmet need for novel therapies. Clinical stage anti-GPC-1 antibody Miltuximab^®^ has proven safe in a first in human study. There is clinical rationale for the therapeutic use of a BiTE construct in PCa [9]. Thus, we engineered a BiTE construct using the GPC-1 binding sequence of Miltuximab^®^ and demonstrated effective and specific T cell activation and PCa cell lysis in two phenotypically distinct GPC-1^high^ PCa cell lines, namely DU-145 and PC3 (derived from a PCa brain metastasis, and bone metastasis, respectively). Importantly, the affinity of MIL-38-CD3 for CD3 was 1000 fold lower than for GPC-1, an important feature for a BiTE, to minimise T cell binding in the absence of tumour cell recognition, and resultant toxicity [24]. These data support the clinical development of MIL-38-CD3 for PCa. Potentially promoting an inflammatory environment in the tumour, we report the release of inflammatory cytokines TNF and IFN-γ from the T cell population. This activated population up-regulated PD-1, suggestive of potential for combination therapies with checkpoint inhibition.

Prostate cancer is considered an “immunologically cold” i.e. not T cell-inflamed, tumour, with few infiltrating immune cells and a paucity of inflammatory cytokines in the local tumour environment [25]. A down-regulation in MHC I expression has been reported in PCa cell lines as well as clinical specimens [26, 27]. Thus, checkpoint inhibitor molecules, have, historically been ineffective in the therapy of PCa in a majority of patients [23]. The use of a BiTE overcomes these obstacles, driving T cell activation without requirement for tumour specific antigen priming [28], while increasing T cell derived local inflammatory cytokine levels such as TNF and IFN-y to support an inflammatory environment and drive further T cell homing [21], local immune cell activation and increased MHC I expression [22]. Here, we demonstrate the potential of a GPC-1 targeted BiTE to achieve this goal. There is clear potential for the use of BiTE constructs as therapeutics for PCa [9], but one wonders whether the combination of checkpoint inhibition with BiTE therapy could offer improved efficacy, and provide some clinical utility for checkpoint inhibitors in this traditionally non-responsive cancer type. This idea of combining checkpoint inhibition with BiTE therapy has been tested preclinically in many studies, demonstrating synergy between checkpoint inhibition and BiTE therapy [28]. In the current study, we found an up-regulation of PD-1 on the surface of MIL-38-CD3 activated T cells, suggesting that a PD-1 inhibitor could extend duration of T cell activation, ultimately improving efficacy. Moreover, the inflammatory environment induced by MIL-38-CD3 at the tumour site may enhance the activity of a checkpoint inhibitor.

The current study aimed to evaluate the ability of MIL-38-CD3 to mediate PCa cell death in vitro. For these studies, two phenotypically distinct PCa cell lines were chosen as standard PCa cell lines used in the field to model PCa in vitro, the DU-145 and PC3 cell lines [19]. Both are androgen independent lines, the DU-145 cell line is derived from a brain metastatic site considered similar in phenotype to adenocarcinoma, while the PC3 line is derived from bone metastasis, similar to the more aggressive prostatic small cell neuroendocrine carcinoma [20, 29]. The comparison was also of interest as the two lines differ significantly in GPC-1 expression, with the highest expression in DU-145, and moderate expression in PC3. We demonstrated the efficacy of MIL-38-CD3 in both cell lines, in terms of T cell activation, cytokine release and cell killing, with significant cell killing achieved at 100 ng/ml for both lines. Of course, the use of primary prostate tumour cells in these in vitro assays would also be informative, particularly given the potential heterogeneity of GPC-1 expression in primary tumour. Moreover, being an in vitro study, one must note the potential influence of the in vivo tumour microenvironment on the apoptosis achieved using MIL-38-CD3 redirected T cells, notably any immunosuppressive factors present, and stromal cell support of cancer cell survival. Indeed, amongst the milieu of influencing factors present in the TME, it is known that prosate stromal cells may support the growth and inhibit apoptosis of prostate tumour cells [30, 31]. This question could be addressed in part using a co-culture of primary stromal cells with primary prostate cells or prostate organoids, isolated from clinical samples [30, 32].

To address the question of the effect of tumour microenvironment on the efficacy of MIL-38-CD3 as well as the next step in clinical development of MIL-38-CD3, we plan to test the construct in an animal model of PCa. A specific challenge in immunotherapeutic validation is the development of a clinically-relevant model. The CD3 arm of the construct does not cross-react with mouse CD3, so human T cells are required. The MIL-38 arm of MIL-38-CD3 does not cross-react with mouse GPC-1. Thus, the simplest model will be engraftment of enriched human T cells together with human tumour cells to establish a xenograft, followed by delivery of MIL-38-CD3 [33–35]. Perhaps more clinically relevant, however, would be a mouse with humanised immune system and subcutaneous PCa xenograft. These studies are currently in planning. Despite not yet having demonstrated efficacy in an in vivo preclinical model, the in vitro validations described in this report strongly suggest the potential of MIL-38-CD3 BiTE as a promising new option in the therapy of PCa, either alone or in combination with checkpoint inhibitor therapy.

The expression of GPC-1 is not limited to PCa, rather, expression of GPC-1 has been described in a variety of solid tumours potentially responsive to BiTE therapy. Protein expression of GPC-1 has been described in breast [36], pancreatic [37, 38], bladder [13], cervical [39] and oesophageal [16] cancers and glioblastoma [40]. We envisage the potential use of MIL-38-CD3 in several solid tumours where it may prove to be efficacious in making known “cold” tumors like prostate and pancreas “hot” and, so, potentially responsive to therapeutic checkpoint inhibition.

## Conclusions

Here, we characterise in vitro the functionality of a GPC-1 directed BiTE in PCa. MIL-38-CD3, based on the clinical antibody Miltuximab^®^, specifically activates and redirects T cells to lyse PCa tumour cells in vitro. We propose a potential for combination therapy of MIL-38-CD3 with checkpoint inhibition, demonstrating PD-1 up-regulation on the surface of MIL-38-CD3 activated T cells. These data collectively support the further clinical development of MIL-38-CD3.

## Supplementary Information

**Additional file 1: Supplementary Figure 1**. Glypican-1 antigen density analysis for cell lines. The density of GPC-1 on the cell surface of a) DU-145 b) Raji c) C3 and d) PC3 was measured using a quantitative flow cytometry assay with MIL-38 as the antibody.

**Additional file 2: Supplementary Figure 2**. Activation of T cells using a non targeted BiTE. Activation of T cells in the presence of DU-145 tumour cells and a non targeted BITE CD3-PEG was used to assess specificity of MIL-38-CD3 BiTE T cell activation. Expression of CD69 was measured by flow cytometry within the CD3^+^CD4^+^ and CD3^+^CD8^+^ T cell gates. Data show combined measurements for 3–4 donors.

**Additional file 3: Supplementary Figure 3**. Expression of GPC-1 in peripheral blood T cells. Anti-GPC-1 antibody Miltuximab^®^ (20 μg/ml) was used to measure expression of GPC-1 in T cells (CD3^+^) by flow cytometry. Detection was achieved using anti-human IgG Alexa fluor 488. The grey filled histogram is secondary only control, the solid black overlay is Miltuximab^®^ staining.

**Additional file 4: Supplementary Figure 4**. Binding of MIL-38-CD3 to, and mediation of T cell cytokine release by T cells cultured with PC3 cells. A. Binding of MIL-38-CD3 was assessed in PC3 cells by flow cytometry. B. The release of IFN-y from T cells cultured with MIL-38-CD3 or control antibody CD3-PEG and PC3 cells was measured by ELISA. *P* values *p < 0.02; **p < 0.001.

## Data Availability

The datasets supporting the conclusions of this article are included within the article (and its additional files).

## Abbreviations

PCa: Prostate Cancer
mCRPC: Metastatic Castrate Resistant Prostate Cancer
GPC-1: Glypican-1
scFv: Single Chain Fragment Variable
BiTE: Bispecific T Cell Engager
RPMI: Roswell Park Memorial Institute
RT: Room Temperature
FBS: Foetal Bovine Serum
HI FBS: Heat Inactivated Foetal Bovine Serum
CHO: Chinese Hamster Ovary
FSW: Flow Cytometry Staining Wash
PBMC: Peripheral Blood Mononuclear Cell
