# Supplementary figures and images for: A bispecific T cell engager targeting Glypican-1 redirects T cell cytolytic activity to kill prostate cancer cells
Source: BMC Cancer. 2020 Dec 10;20:1214. doi: 10.1186/s12885-020-07562-1 (PMC7727117; doi:10.1186/s12885-020-07562-1)

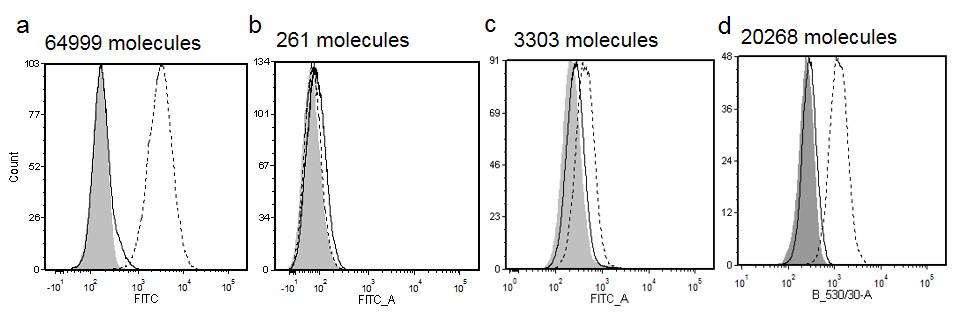

Supplement: Supplementary file 1 — Additional file 1: Supplementary Figure 1. Glypican-1 antigen density analysis for cell lines. The density of GPC-1 on the cell surface of a) DU-145 b) Raji c) C3 and d) PC3 was measured using a quantitative flow cytometry assay with MIL-38 as the antibody. [file 12885_2020_7562_MOESM1_ESM.jpg]

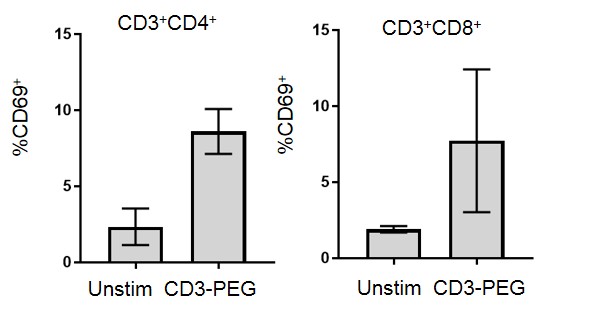

Supplement: Supplementary file 2 — Additional file 2: Supplementary Figure 2. Activation of T cells using a non targeted BiTE. Activation of T cells in the presence of DU-145 tumour cells and a non targeted BITE CD3-PEG was used to assess specificity of MIL-38-CD3 BiTE T cell activation. Expression of CD69 was measured by flow cytometry within the CD3+CD4+ and CD3+CD8+ T cell gates. Data show combined measurements for 3–4 donors. [file 12885_2020_7562_MOESM2_ESM.jpg]

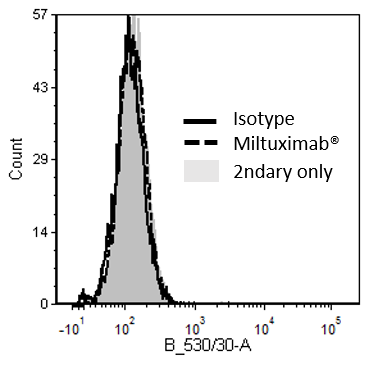

Supplement: Supplementary file 3 — Additional file 3: Supplementary Figure 3. Expression of GPC-1 in peripheral blood T cells. Anti-GPC-1 antibody Miltuximab® (20 μg/ml) was used to measure expression of GPC-1 in T cells (CD3+) by flow cytometry. Detection was achieved using anti-human IgG Alexa fluor 488. The grey filled histogram is secondary only control, the solid black overlay is Miltuximab® staining. [file 12885_2020_7562_MOESM3_ESM.tif]

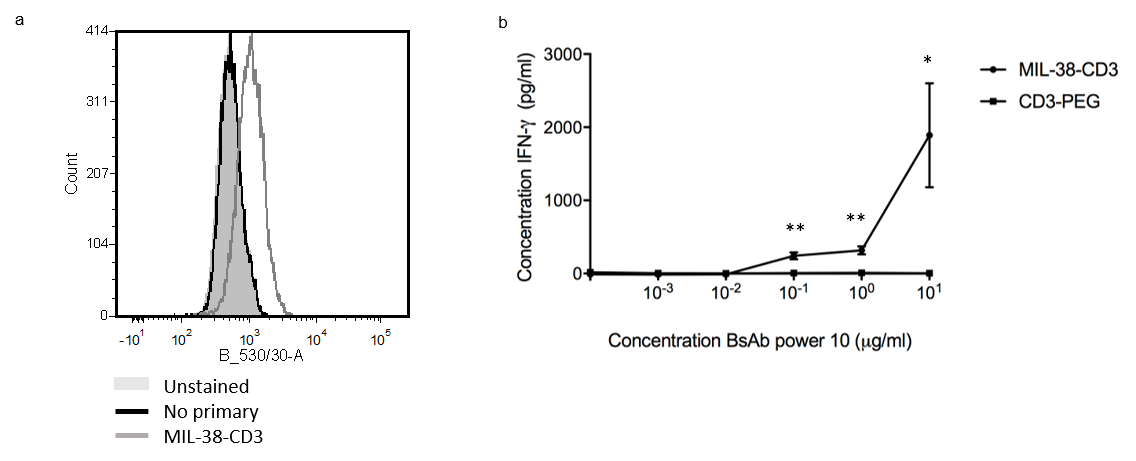

Supplement: Supplementary file 4 — Additional file 4: Supplementary Figure 4. Binding of MIL-38-CD3 to, and mediation of T cell cytokine release by T cells cultured with PC3 cells. A. Binding of MIL-38-CD3 was assessed in PC3 cells by flow cytometry. B. The release of IFN-y from T cells cultured with MIL-38-CD3 or control antibody CD3-PEG and PC3 cells was measured by ELISA. P values *p < 0.02; **p < 0.001. [file 12885_2020_7562_MOESM4_ESM.tif]
